# Treatment preferences in spinal muscular atrophy: A swing weighting study for caregivers of patients with SMA types 1 and 2

**DOI:** 10.1371/journal.pone.0309666

**Published:** 2024-10-21

**Authors:** Anish Patel, Walter Toro, Siobhan Bourke, Yemi Oluboyede, Sylvaine Barbier, Nataliya Bogoeva, Sandra P. Reyna, Omar Dabbous

**Affiliations:** 1 Novartis Gene Therapies, Inc., Bannockburn, IL, United States of America; 2 Putnam Associates, Mayo, Ireland; 3 Putnam Associates, London, United Kingdom; 4 Putnam Associates, Lyon, France; 5 Putnam Associates, Sofia, Bulgaria; Fondazione Policlinico Universitario Gemelli IRCCS, ITALY

## Abstract

Spinal muscular atrophy (SMA) is a genetic neuromuscular disorder characterized by skeletal muscle weakness and atrophy. Patients with SMA types 1 and 2 develop severe disabilities conferring substantial patient and caregiver burden. Caregiver treatment characteristic preferences are useful for informing treatment choices and improving adherence. We aimed to identify drivers of SMA treatment preference from the perspective of caregivers of patients with SMA types 1 or 2 in the United States. We quantified the relative importance of different treatment characteristics and compared preferences for hypothetical treatment scenarios. Treatment attributes and attribute levels elicited were based on a literature search and interviews with caregivers and health care professionals. The most important treatment characteristics from the perspective of health care professionals and caregivers were identified and used in a survey to quantify relative importance for caregivers. Caregivers completed surveys regarding their preferences using swing weighting methodology. These results were used to estimate the relative value of four hypothetical SMA treatment scenarios exploring different modes of treatment administration. The swing weighting survey, completed by 20 caregivers, demonstrated that the attributes driving treatment preference were reduction in permanent ventilation needs and risk of severe adverse events, followed by treatment access (including cost coverage and availability), increased ability to sit without support, and less treatment administration burden. The hypothetical SMA treatment scenarios with the highest relative value offered an easier mode of administration, lowest risk of severe adverse events, less need of permanent ventilation, and highest ability of patients to feed and sit without support. Our findings suggest that caregivers prefer a treatment with reduced clinical burden and risk in which the cost is covered and treatment is available in the short term. These results can provide important contextual information for decision-makers and help promote patient-centered care for patients with SMA.

## Background

Spinal muscular atrophy (SMA) is a rare genetic disorder characterized by weakness and atrophy of skeletal muscles caused by deletions or mutations in the *survival motor neuron 1* (*SMN1*) gene and the presence of *SMN2* [1, 2]. Progressive muscle weakness and difficulties with swallowing and breathing appear due to the loss of motor neurons and ultimately lead to death without treatment [2, 3]. SMA is categorized into five clinical subgroups from 0 (most severe) to 4 (least severe) based on age of onset and severity of symptoms [4]. Children with SMA type 1 (age of onset: <6 months) have breathing and swallowing problems that may lead to choking or gagging, and most of them are unable to support their head or sit unassisted. SMA type 1 has a prevalence of approximately 1 to 2 in 100,000, with an incidence close to 1 in 10,000 live births, accounting for approximately 60% of all SMA cases. Patients living with SMA type 1 will become dependent on a mechanical ventilator or even die in the absence of an effective disease-modifying treatment (DMT) [5]. Children with SMA type 2 (age of onset: 6–18 months), representing approximately 20% of new cases, never stand or walk independently, but some patients may be able to stand with assistance [4, 6]. For patients with SMA type 2, the natural disease course is slower compared with SMA type 1, but is still marked by significant accumulating morbidity, with severe disability for patients and tremendous burden placed on their caregivers [7].

Management of SMA requires the dedication of a multidisciplinary team [8]. Such teams will often include neuromuscular consultants (often neurologists), physiotherapists, pulmonologists, orthopedic specialists, and gastroenterologists. Physical and occupational therapy, assistance with speech and swallowing, and respiratory and nutritional support are key elements of assisting patients with SMA to maximize their functional abilities. Mobility issues often require assistive devices or physical therapy regimens. Some individuals with SMA require support for breathing and feeding, as well as pain management. Nearly all patients with SMA also report the dual needs of managing fatigue and maintaining stamina.

Three DMTs have been approved for SMA in recent years: an *SMN1* gene replacement therapy designed to deliver a functional copy of the gene encoding human SMN protein, onasemnogene abeparvovec (ZOLGENSMA); an *SMN2*-directed antisense oligonucleotide, nusinersen (SPINRAZA); and an *SMN2* gene-splicing modifier, risdiplam (EVRYSDI) [9–11]. These DMTs answer an unmet need for treatments that improve motor function and life expectancy for patients with SMA [12].

Considering the disease burden of SMA, the benefits and limitations of the currently available DMTs, and the evolving treatment landscape, it is crucial to understand how treatment characteristics may influence caregiver decisions [13]. Our study aims to explore the drivers of preference for SMA treatment from the perspective of caregivers of patients with SMA types 1 or 2 in the United States.

## Methods

### Study design and participants

An online, cross-sectional, interviewer-assisted, swing weighting (SW) survey was designed and employed to estimate caregivers’ preferences concerning SMA treatments for children with SMA types 1 or 2. SW describes the relative importance of the improvement of an objective’s attribute (i.e., SMA treatment attributes in this study), from its worst possible to best possible attribute level, compared with the improvement from worst to best of the other attributes [14]. These results are reliable and comparable to results of a discrete choice experiment (DCE) [15]. SW directly captures preferences within the elicitation task rather than within a separate utility estimate, thus permitting the generation of valid utility differences with limited sample size if the population is sufficiently homogenous [15]. Given the expected low sample size for survey recruitment, an SW survey was preferable over a DCE.

The inclusion criteria for the survey respondents included parents or caregivers who were the primary caregiver of an individual diagnosed with SMA type 1 or type 2 by a clinician and who was not currently staying in an inpatient facility. More than one parent or caregiver per family could complete the survey. Participants who cared for more than one patient with SMA provided demographic data for all patients in their care. For all other survey questions, respondents provided one response regardless of the number of patients with SMA in their care.

Ethical approval for the study protocol and participant-facing documents were received by an independent international US-based institutional review board (Advarra) and an independent ethics reviewer in the United Kingdom. All participants provided electronic consent prior to starting the online survey and were compensated for their time with a $65 USD Rybbon gift card.

### SW survey development

**Fig 1:**
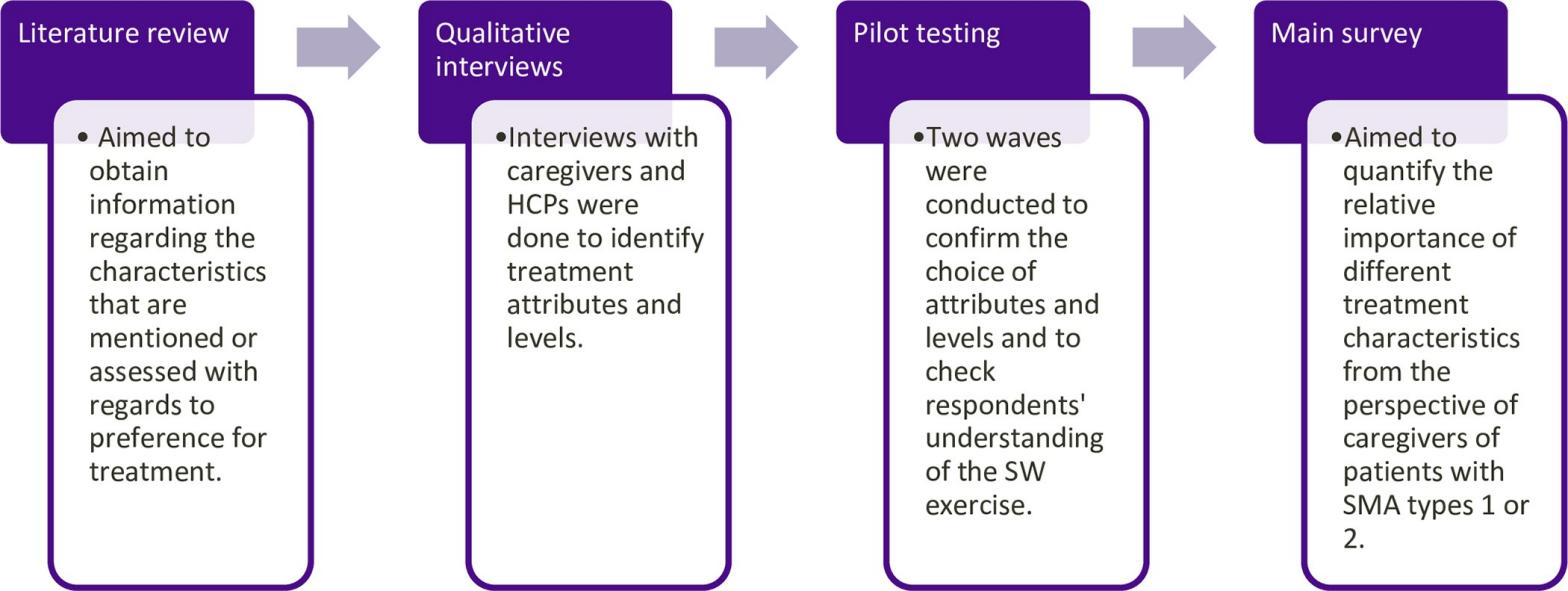
Stages of SW survey development and conduct. HCP, health care provider; SMA, spinal muscular atrophy; SW, swing weighting.

The survey was constructed in Qualtrics (Provo, UT) and responses were required for all questions to not allow for missing data. The stages of the SW survey development are presented in **Fig 1**. The attributes related to SMA treatment characteristics included in the survey were first identified from a targeted literature search conducted on 9 April 2019 via the Medline database (access via OVID interface; with limit on last 10 years). This search was designed to identify clinical studies that examined patient satisfaction or preferences in the context of treatment for neuromuscular or motor neuron conditions. Interviews were then conducted between February and April 2021 with four caregivers of children (aged 3–15 years) who received SMA DMTs and four health care professionals (HCPs; three neurologists and one nurse specializing in pediatric neurology) to ensure the attributes identified from the targeted literature search were salient to patients and their caregivers. After eight interviews, no new attributes were introduced or discussed by the participants, thus data saturation was deemed to be achieved.

The attribute levels (lower, middle, and upper) were selected with the goal of representing credible attribute levels that can be experienced by the target population. The lower and upper attribute levels were selected based on the range of outcomes in clinical studies identified in the literature review, as this would demonstrate the range of outcomes that patients might expect. The middle attribute level assumed linear utility; thus the interim level was calculated as the mid-value of the upper and lower levels. **Table 1** contains the final list of attributes and associated levels included in the SW survey.

**Table 1:** Attributes and attribute levels included in the SW exercise.

Attribute	Attribute description	Attribute levels	Attribute level source
Attributes with numerically ranked levels*			
**Ability to sit without support after 1 year of treatment**	The percentage of patients who start treatment at diagnosis and are able to sit without support after 1 year of treatment	Lower: 30% of patients	FIREFISH part 2 (NCT02913482) [16]
		Middle: 45% of patients	Median of lower and upper levels
		Upper: 60% of patients	STRIVE US Complete (NCT03306277) [17]
**Need for permanent ventilation after 1 year of treatment**	The percentage of patients who start treatment at diagnosis and spend more than 16 hours a day on ventilatory support after 1 year of treatment	Lower: 40% of patients	ENDEAR (NCT02193074) [18]
		Middle: 25% of patients	Median of lower and upper levels
		Upper: 10% of patients	STRIVE US Complete (NCT03306277) [17]
**Ability to feed orally after 1 year of treatment**	The percentage of patients who start treatment at diagnosis and are able to eat at least some part of their food (liquid or solid) orally after 1 year of treatment	Lower: 40% of patients	STRIVE US Complete (NCT03306277) [17]
		Middle: 60% of patients	Median of lower and upper levels
		Upper: 80% of patients	FIREFISH part 2 (NCT02913482) [16]
**Risk of severe adverse events within 1 year after treatment initiation**	The risk that the patient experiences severe adverse events (e.g., liver damage, kidney damage, or pneumonia) which require hospitalization, result in persistent or significant disability, or are life-threatening within 1 year after treatment initiation	Lower: 50% of patients	Hypothetical
		Middle: 25% of patients	FIREFISH part 2 (NCT02913482) [16]
		Upper: 10% of patients	STRIVE US Complete (NCT03306277) [17]
**Attributes with non-numerically ranked levels**			
**Burden of treatment administration**	The invasiveness and the frequency of the treatment administration, risk of adverse events, and laboratory testing requirements	Six spinal injections first year, three in every following year (continuously; administered under local anesthesia for 3–6 hours in a medical center; headache and infection at injection site are common; blood and urine tests are needed before each administration)	Nusinersen prescribing information [9]
		One-time spinal injection for lifetime (administered under local anesthesia for 3–6 hours in a medical center; headache and infection at injection site are common; blood test is needed before treatment administration and every 2 weeks for 3 months following administration)	Hypothetical
		One-time intravenous infusion for lifetime (no anesthesia is needed; 3 hours in a medical center; blood test is needed for treatment administration and every 2 weeks for 3 months following administration)	Onasemnogene abeparvovec prescribing information [10]
		Daily oral solution (continuously; administered via a feeding tube or by mouth; no laboratory tests are needed)	Risdiplam prescribing information [11]
**Access to treatment**	Treatment cost coverage (through private health insurance, public health plan, or patient support program) and time to treatment availability based on financing or payment agreement terms	Treatment is covered and available after 1-week financing procedure	Based on targeted literature search and interviews with caregivers and HCPs
		Treatment is covered and available after 4-week financing procedure	
		Treatment is not covered and available immediately after agreeing to an arrangement to pay within 1 year	
		Treatment is not covered and available immediately after agreeing to an arrangement to pay within 5 years	
**Type of facility in which the treatment is available**	Type of medical facility for SMA treatment availability and the typical location relative to patient’s home	Any neurologic department or office that offers SMA care (usually closer to patient’s home)	Based on targeted literature search and interviews with caregivers and HCPs
		Highly specialized neuromuscular facility (usually farther from patient’s home)	

HCP, health care professional; SMA, spinal muscular atrophy.

*Upper, middle, and lower levels represent the best to worst clinical outcomes, respectively (i.e., a greater percentage of patients able to sit independently or eat orally and a lower percentage of patients requiring permanent ventilation and at risk for serious adverse events within 1 year after treatment initiation.

A pilot of the SW survey was completed by the caregivers with a trained interviewer to confirm the choice of attributes and attribute levels and to ensure respondents understood the attributes and SW exercise prior to commencing the SW survey. This was followed by a cognitive debriefing interview based on an interview guide. Based on the pilot study, an interviewer-assisted online survey was identified as the preferred method of data collection due to issues surrounding respondents’ ability to self-complete. Further details and the results of the attribute elicitation phase and pilot testing can be found in **Appendix 1 in S1 File**.

## SW exercise

**Fig 2:**
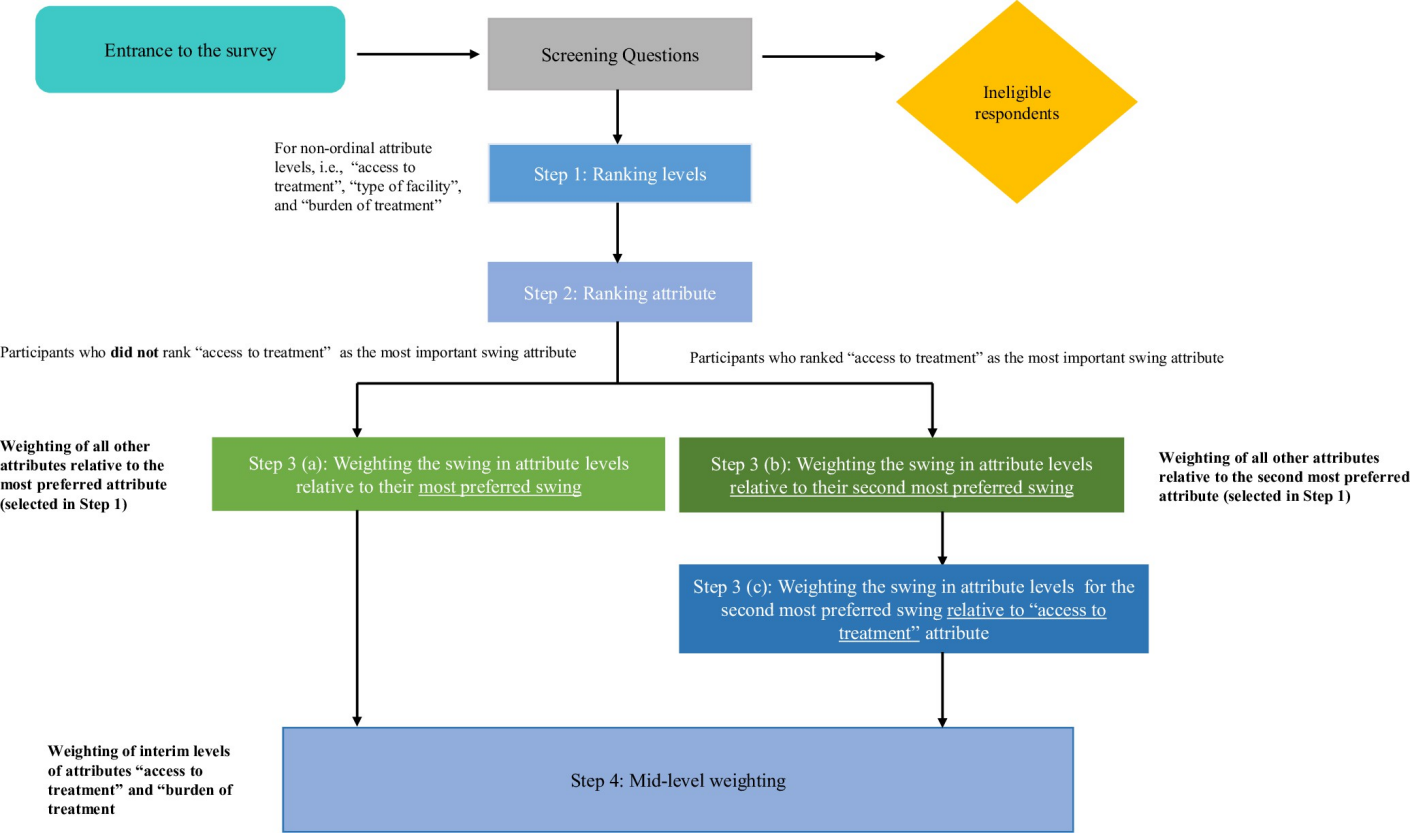
Survey details.

Preference surveys can be challenging to design and present questions so that respondents can understand and make complex choices. Ensuring that the design and the questionnaire were engaging and sufficiently complex when describing the treatment options without the survey being cognitively burdensome was a particular challenge in this study, especially around the context of “availability of treatment.” The approach undertaken by the research team was a “caregiver first approach,” meaning we strived to include attributes that were defined as important to caregivers. However, the inclusion of the “availability of treatment” attribute required some modification to the survey design as we wanted to ensure that it was included but did not become the focal point of the preference study. This modification was also undertaken to ensure that variation in coverage of the treatment or type of insurance that the participant had in real life did not impact the results of the survey. In addition, the pilot survey was found to be difficult to complete as respondents struggled and exhibited some decision heuristics with the valuation of the levels. Respondents applied valuations based on the linear nature of the attribute, and thus some complexities were removed in favor of simplification of the survey design in the section valuing the mid-level of the attributes. The survey approach undertaken is detailed in **Fig 2** and described below.

**Fig 3:**
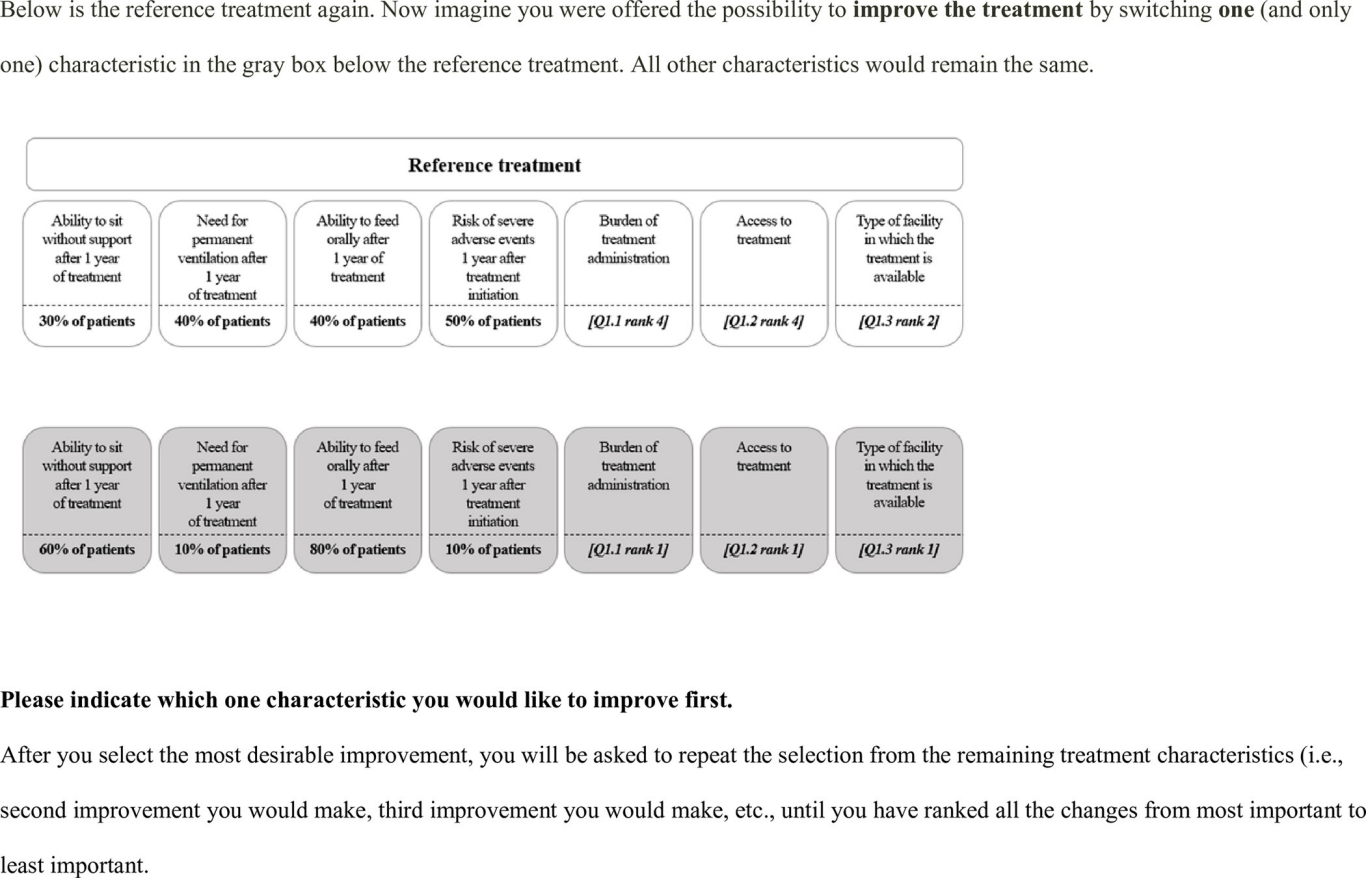
Ranking of attributes.

All participants were presented with detailed descriptions of the attributes and attribute levels to be considered in the preference study. The swing weighting exercise comprised four steps:

1. Attribute level ranking for non-ordinal attribute levels (“access to treatment,” “type of facility,” and “burden of treatment”): Participants were not asked to rank attribute levels for attributes with numerical levels as the ordinal ranking provided was assumed to be correct.
2. Attribute ranking: Participants were introduced to a reference treatment in which all criteria were set to the worst possible attribute levels either assumed or identified during the attribute level ranking step. Respondents were then asked to indicate which attribute from a provided list they wanted to improve (i.e., to “swing”) from the worst to best level first (**Fig 3**). After making this choice, participants were then asked to indicate which attribute was the second attribute they wanted to improve from the worst to best level. This was repeated until all attributes were ranked. After ranking all attributes, participants were asked to confirm their ranking and were given the opportunity to adjust their selection if necessary.
3. (a) Scoring or weighing the “swing” of each attribute relative to the most preferred attribute “swing”: Participants were asked to assign points between 0 (least important/worst attribute) and 100 (most important/best attribute) to each attribute change they had ranked in the attribute ranking step. The first ranked attribute was automatically assigned a score of 100; participants were then asked to assign points to all other attributes relative to this 100-point benchmark. The purpose of this weighting step was to determine the relative importance of swings in criteria and to elicit participants’ preferences between criteria as “trade-offs.”
3. (b) If participants selected “access to treatment” as their most preferred ranking, the “swing” of each attribute was scored relative to their second most preferred attribute “swing.” Similar to participants in step 3a, participants were then asked to assign points between 0 (least important/worst attribute) and 100 (most important/best attribute). In this case, 100 points were assigned to the second most preferred attribute and again, participants were asked to score the attributes relative to the attribute that was scored 100 points. The “access to treatment attribute” was excluded from this step and was valued in step 3(c).
3. (c) If participants selected “access to treatment” as their most preferred attribute, they were asked to score or weight the “swing” of their most preferred attribute “access to treatment to their second most preferred attribute “swing.” Here the “access to treatment” attribute was scored at 100 points with the second most preferred attribute being scored relative to this.
4. To simplify the estimation of mid-level utilities and as observed in the pilot survey, participants followed the assumption of linearity in the attribute levels responses. Therefore, participants were not required to value those attributes that had an ordinal ranking. Thus, mid-level weight estimation was only completed by the respondents for “burden of treatment administration” and “access to treatment.” Here, participants valued the mid-level with the score being relative to = 0 (worst level) to 100 (best level) according.

To orient the participants to the study, they were first presented an example study about preferences for car attributes (types, color, transmission) that followed the structure of the SW exercise.

### Relative value of hypothetical SMA treatment scenarios

The results of the SW exercise were used to evaluate the relative value of four hypothetical SMA treatment scenarios which allowed for the estimate of the caregiver perceived value for new modalities of treatment administration and of the impact of subsequent treatments. The description of the hypothetical treatments was decided by the research team. These hypothetical treatments, which were created to demonstrate a range of treatment preferences rather than to identify a current treatment, were developed as follows:

- Treatment A: Best treatment as described by the perceived “best” attribute levels according to the SW exercise
- Treatment B: Worst treatment as described by the perceived “worst” attribute levels according to the SW exercise
- Treatment C: Average treatment as described by those that were perceived as “middle” attribute levels according to the SW exercise
- Treatment D: Same as Treatment A but with a change in mode of treatment administration

### Statistical analysis

Descriptive statistics were used to describe, summarize, and graphically represent caregivers’ sociodemographic details and patients’ clinical backgrounds. Analysis of attributes and attribute level ranking was completed by examining frequencies. The SW exercise results were described by the mean and standard deviation (SD) of the amount of change in one attribute (from worst to best level) that would cause survey respondents to be indifferent between that change and a change in another attribute.

**Fig 4:**
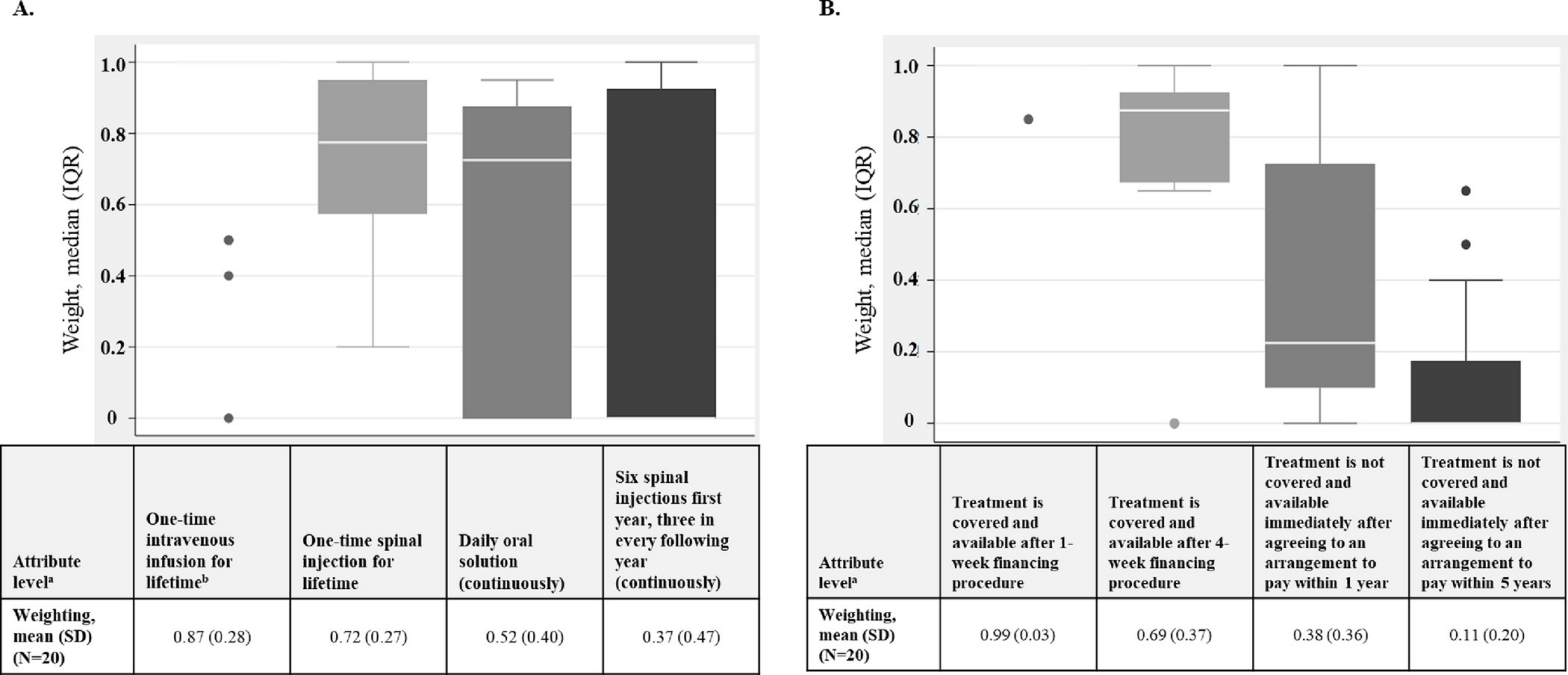
Weight distribution associated with attribute levels for the “access to treatment” attribute.

For participants who did not rank “access to treatment” as the most important attribute (**Fig 4**):

- A swing from the worst to the best attribute level was assigned f_1_ = 100 points (i.e., the top attribute [denoted with ranking index 1] was assigned 100 points)
- Then each attribute was assigned a score f_i_ relative to the top attribute (directly obtained from rating scale), between 0 and 100.

To account for the modified survey design used when “access to treatment” was chosen as the most important attribute, the “access to treatment” attribute was assigned a score of f_1_ = 100 points. The second most important attribute was then assigned a score of f_2_.

All other attributes were rated by participants on a scale where the second most important attribute had a score of 100 and needed to be rescaled for each participant as follows:

$$f_{i}=\left(\frac{S_{i}}{f2}\right)*100$$

in which s_i_ represents the score directly provided in the questionnaire and f_2_ is the score assigned to the most important attribute. The presence of attribute heterogeneity was examined using the ratio of SD to the mean [19] with a score greater than 100% indicating that the variations in preferences are high and thus indicating higher heterogenity.

To estimate the partial value function (mid-level value) for “burden of treatment administration” and “access to treatment” attribute levels, data were normalized to constrain the attribute levels on a 0–1 scale, where 0 was the worst and 1 was the best attribute level. Participants assigned a score v_j_ for these attributes between 0 and 100 to each attribute level. The partial value function for each participant and for each attribute level takes the value:

$$\frac{vj}{100}$$

For the “type of facility in which the treatment is available” attribute, there were only two levels; the preferred level was assigned a value of 1 and the other a value of 0.

The remaining attributes were not presented to the participants and instead ratio values were calculated. For the “ability to sit without support after 1 year of treatment,” “need for permanent ventilation after 1 year of treatment,” and “ability to feed orally after 1 year of treatment” attributes, a ratio value was calculated as follows:

- (Intermediate level–Worst level)/(Best level–Worst level)

For the “risk of severe adverse events within 1 year after treatment initiation” attribute, the mid-level (25% of people with SMA) was estimated at 0.6.

To estimate the relative value of hypothetical SMA treatment scenarios, a matrix was created, and criteria were listed for each hypothetical therapy. If a feature of the hypothetical treatment was present, the attribute weight was assigned. For each hypothetical treatment scenario, the scores of the alternative value on each criterion with the weight of that criterion were multiplied and summed across *all* criteria. After eliciting the scores and weights, the aggregation was performed using an additive model:

$$u=\sum_{i=1}^{7}w_{i}.v_{j_{i}}$$

in which *j_i_* represents the level of each attribute i (so $v_{j_{i}}$ is the value of the partial value function for attribute i, given that this attribute has the level j_i_) [12].

## Results

### Demographics

Twenty caregivers of patients with SMA types 1 or 2 participated in the study (**Table 2**). In total, 16 participants (80%) were female, with a mean (SD; range) age of 38 (7; 27–53) years. The majority of participants (90%) cared for only one child with SMA. Nine participants (45%) stated that they were full-time caregivers. For those employed outside of caregiving, seven (35%) were employed full time, one (5%) was self-employed, and one (5%) was employed part time; two (10%) were unemployed. The most common insurance type for children with SMA represented in the study was Medicaid (12/20; 60%), followed by employer-based private health insurance (4/20; 20%). Respondents most commonly had a master’s degree (11/20; 55%) or a bachelor’s degree (8/40; 40%). The most common geographic location for living was in a suburb near a large city (9/20; 45%).

**Table 2:** Caregiver demographics.

	Caregivers (N = 20)
**Age, years**	
Mean (SD)	38 (7)
Range	27–53
**Sex, n (%)**	
Female	16 (80)
Male	4 (20)
**Employment, n (%)**	
Full-time caregiver	9 (45)
Employed full time	7 (35)
Unemployed	2 (10)
Employed part time	1 (5)
Self-employed/business owner	1 (5)
**Insurance, n (%)**	
Medicaid	12 (60)
Employer-based private insurance	4 (20)
Medicare	2 (10)
No medical insurance	1 (5)
Other	1 (5)
**Education, n (%)**	
Master’s degree or higher	11 (55)
Bachelor’s degree	8 (40)
Did not graduate high school	1 (5)
**Location, n (%)**	
Suburb near a large city	9 (45)
Small city or town	2 (25)
Large city	4 (20)
Rural area	2 (10)
**Number of children with SMA, n (%)**	
1	18 (90)
>1*	2 (10)

SMA, spinal muscular atrophy.

*If caregivers had more than one child under their care, they answered the demographic and clinical characteristics of their youngest child with SMA type 1 or SMA type 2.

The majority (15/20; 75%) of patients had SMA type 1, with an even split between sexes (10/20; 50% each) (**Table 3**). The sample reported the most advanced motor activity currently achieved by a child was standing with support (7/20; 35%), followed by sitting independently for >10 seconds (4/20; 20%), and head control and walking without support (3/20); 15% each). All children with SMA were receiving at least one DMT for SMA.

**Table 3:** Demographics and clinical characteristics of children with SMA.

	Children with SMA (N = 20)
**SMA type, n (%)**	
1	15 (75)
2	5 (25)
**Age, months**	
Mean (SD)	11 (5)
Range	4–21
**Sex, n (%)**	
Male	10 (50)
Female	10 (50)
**Motor function, n (%)** ^*^	
Head control	3 (15)
Sitting independently >10 seconds	4 (20)
Standing with support	7 (35)
Walking with support	2 (10)
Walking without support	3 (15)
None of the above	1 (5)
**Requires ventilation, n (%)** ^*^	
Yes, non-invasive	1 (5)
Yes, invasive	3 (15)
No ventilation	16 (80)
**Independent oral feeding, n (%)** ^*^	
Yes, all the food	12 (60)
Yes, most of the food	1 (5)
Some, some of the food	3 (15)
None	4 (20)
**Number of DMTs received/receiving, n (%)**	
1	8 (40)
2	11 (55)
≥3	1 (5)

DMT, disease-modifying treatment; SD, standard deviation; SMA, spinal muscular atrophy.

*Listed in order of increasing motor function, respiratory, and feeding abilities.

### Ranking of non-numerical attribute levels

For the “burden of treatment administration” attribute, the “a one-time intravenous infusion for lifetime” attribute level was most preferred (**Table 4**). The least preferred attribute level was “six spinal injections first year, three in every following year.” For the “access to treatment” attribute, “treatment is covered by insurance and available after a 1-week financing procedure” was ranked as best, whereas the two attributes detailing “treatment is not covered” were ranked equally as the worst. Finally, for the “type of facility in which the treatment is available,” “any neurologic department or office that offers SMA care (usually closer to patient’s home)” was preferred to a “highly specialized neuromuscular facility (usually farther from the patient’s home)”.

**Table 4:** Attribute level ranking of non-numerical attributes.

Attribute	Attribute level ranking (N = 20)		
	Rank	Attribute level	Participants selecting this rank, n (%)*
Burden of treatment administration^†^	1	One-time intravenous infusion for lifetime	16 (80)
	2	One-time spinal injection for lifetime	15 (40)
	3	Daily oral solution (continuously)	11 (55)
	4	Six spinal injections first year, three in every following year (continuously)	12 (60)
Access to treatment	1	Treatment is covered and available after 1-week financing procedure	19 (99)
	2	Treatment is covered and available after 4-week financing procedure	15 (75)
	3	Treatment is not covered and available immediately after agreeing to an arrangement to pay within 5 years	13 (65)
	4	Treatment is not covered and available immediately after agreeing to an arrangement to pay within 1 year	13 (65)
Type of facility in which treatment is available	1	Any neurologic department or office that offers SMA care (usually closer to patient’s home)	12 (60)
	2	Highly specialized neuromuscular facility (usually farther from patient’s home)	8 (40)

SMA, spinal muscular atrophy.

*Ranking based on frequency.

^†^Full level descriptions for this attribute are provided in **Table 1**.

### Attribute ranking and weighting

The “risk of severe adverse events within 1 year after treatment initiation” was ranked as the most important attribute for change with a swing from lower (50% of patients) to upper (10% of patients) levels (**Table 5**). The second most important attribute for change was “need for permanent ventilation after 1 year of treatment” with a swing from lower (40% of patients) to upper (10% of patients) levels. “Access to treatment” was ranked third and “burden of treatment administration” and “ability to sit without support after 1 year of treatment” were equally ranked fourth. The “ability to feed orally after 1 year of treatment,” followed by the “type of facility in which treatment is available” were ranked as the two least important attributes.

**Table 5:** Attribute ranking and weighting.

Attribute	Attribute Ranking (N = 20)			Attribute Weighting (N = 20)		
	Rank	Swing	Participants selecting this rank, n (%)*	Rank	Weight, mean (SD; range)	Ranking heterogeneity
Risk of severe adverse events within 1 year after treatment initiation	1	Lower (50% of patients) to upper (10% of patients) levels	6 (30)	2	0.188 (0.06; 0.08–0.32)	32.9%
Need permanent ventilation after 1 year of treatment	2	Lower (40% of patients) to upper (10% of patients) levels	7 (35)	1	0.194 (0.5; 0.07–0.26)	24.9%
Access to treatment	3	Worst to best level varied	6 (30)	3	0.155 (0.069; 0.00–0.253)	44.5%
Burden of treatment administration	4^†^	Worst to best level varied	5 (25)	4^†^	0.145 (0.069; 0–0.345)	50.1%
Ability to sit without support after 1 year of treatment	4^†^	Lower (30% of patients) to upper (60% of patients) levels	5 (25)	4^†^	0.145 (0.048; 0.049–0.231)	33.4%
Ability to feed orally after 1 year of treatment	6	Lower (40% of patients) to middle (60% of patients) levels	9 (45)	6	0.130 (0.05; 0.05–0.26)	39.8%
Type of facility in which treatment is available	7	Worst to best level varied	17 (85)	7	0.042 (0.038; 0.00–0.179)	88.7%

SD, standard deviation.

Attributes ordered by ranking from most to least important.

*Ranking based on frequency.

^†^Level 5 ranking does not exist because two attributes were equally ranked in the fourth position.

In terms of attribute weighting, the “need for permanent ventilation after 1 year of treatment” was rated highest (mean weight: 0.194), followed by the “risk of severe adverse events within 1 year after treatment initiation” (mean weight: 0.188; **Table 5**). Though the ranking and weighting order of these two attributes differed, the difference in attribute mean weights was small (0.006). The weighting of the remaining attributes matched the same order observed in the ranking exercise. Rating heterogeneity was highest for the “type of facility in which the treatment is available,” followed by “burden of treatment administration,” “access to treatment,” and “ability to feed orally after 1 year of treatment.” The least heterogeneity was observed in the “need for permanent ventilation after 1 year of treatment”.

Heterogeneity scores were less than the threshold of variation (100%). The highest heterogeneity (88.7%) was observed for the “type of facility in which the treatment was available” with the least heterogeneity (24.9%) observed in the attribute “need for permanent ventilation after 1 year of treatment”.

### Mid-level weighting

For the “burden of treatment administration” attribute, the one-time administration routes were preferred to daily oral administration or multiple spinal injections (**Table 6**). The most preferred level was the “one-time intravenous infusion for lifetime,” which was preferred over a “one-time spinal injection for lifetime.” For the continuous treatment levels, the “daily oral solution (continuously)” level was preferred over the “six spinal injections first year, three in every following year (continuously)” level.

**Table 6:** Mid-level weighting.

Attribute(s)	Attribute level*	Weighting, mean (SD) (N = 20)
Burden of treatment administration	One-time intravenous infusion for lifetime	0.87 (0.28)
	One-time spinal injection for lifetime	0.72 (0.27)
	Six spinal injections first year, three in every following year (continuously)	0.37 (0.47)
	Daily oral solution (continuously)	0.52 (0.40)
Access to treatment	Treatment is covered and available after 1-week financing procedure	0.99 (0.03)
	Treatment is covered and available after 4-week financing procedure	0.69 (0.37)
	Treatment is not covered and available immediately after agreeing to an arrangement to pay within 1 year	0.38 (0.36)
	Treatment is not covered and available immediately after agreeing to an arrangement to pay within 5 years	0.11 (0.20)
Type of facility in which the treatment is available ^†^	Any neurologic department or office that offers SMA care (usually closer to patient’s home)	1 (NA)
	Highly specialized neuromuscular facility (usually farther from patient’s home)	0 (NA)
Ability to sit without support after 1 year of treatment^‡^; Need for permanent ventilation after 1 year of treatment^‡^; Ability to feed orally after 1 year of treatment^‡^	Lower level	0 (NA)
	Middle level	0.5 (NA)
	Upper level	1 (NA)
Risk of severe adverse events within 1 year after treatment initiation^‡^	Lower level	0 (NA)
	Middle level	0.6 (NA)
	Upper level	1 (NA)

NA, not applicable; SD, standard deviation; SMA, spinal muscular atrophy.

*Attribute levels displayed in order of highest to lowest weighting.

^†^Full level descriptions for all attributes are provided in **Table 1**.

^‡^Weights were calculated and not assigned by respondents.

For the “access to treatment” attribute, the levels in which treatment was covered were preferred over the levels in which treatment was not covered (**Table 6 and Fig 3)**. For the levels including treatment coverage, the level with the 1-week financing agreement was preferred over the 4-week financing agreement. The 5-year payment arrangement level was preferred over the 1-year payment arrangement level for levels not including treatment coverage. The distribution of responses was lowest for the most preferred level (“treatment is covered and available after 1-week financing procedure”) and highest for the “treatment is not covered and available immediately after agreeing to an arrangement to pay within 5 years.” The “treatment is not covered and available immediately after agreeing to an arrangement to pay within 1 year” level had the most outliers (n = 2); these are the data points that extend beyond 1.5× the interquartile range. The calculated mid-level weights for all other attributes and their levels are presented in **Table 6**.

### Evaluation of hypothetical SMA treatment scenarios

Based on caregiver preferences in the SW exercise, four hypothetical SMA treatment scenarios were derived (**Table 7**). The results of this study indicated that Treatment Scenario A (including the highest ranked attribute level) was most preferred, followed by Treatment Scenario D (including the same attribute levels as Treatment Scenario A but with a different treatment mode). As expected, Treatment Scenario C (including the middle-ranked attribute levels) followed by Treatment Scenario B (including the lowest ranked attribute levels) were least preferred.

**Table 7:** Evidence matrix for SMA hypothetical treatment scenarios.

Treatment scenario	Scenario description	Attribute and attribute levels* (assigned score per attribute)					Total assigned score for treatment scenario (N = 20)
		Burden of treatment administration	Need for permanent ventilation after 1 year of treatment	Ability to feed orally after 1 year of treatment	Ability to sit without support after 1 year of treatment	Risk of severe adverse events within 1 year after treatment initiation	
Treatment A	Best treatment as described by the perceived “best” attribute levels according to the SW exercise	One-time intravenous infusion for lifetime (0.87)	10% of patients (1)	80% of patients (1)	60% of patients (1)	10% of patients (1)	4.87
Treatment D	Same as treatment A but with a change in mode of treatment administration	One-time spinal injection (0.72)	10% of patients (1)	80% of patients (1)	60% of patients (1)	10% of patients (1)	4.72
Treatment C	Average treatment as described by those that were perceived as “middle” attribute levels according to the SW exercise	Daily oral solution (continuously) (0.52)	25% of patients (0.5)	60% of patients (0.5)	45% of patients (0.5)	25% of patients (0.6)	2.62
Treatment B	Worst treatment as described by the perceived “worst” attribute levels according to the SW exercise	Six spinal injections first year, three in every following year (continuously) (0.37)	40% of patients (0)	40% of patients (0)	30% of patients (0)	50% of patients (0)	0.37

SW, swing weighting.

Treatment scenarios are presented in order of highest to lowest total score.

*Full level descriptions for all attributes are provided in **Table 1**.

## Discussion

Considering the burden SMA confers to both patients and caregivers, as well as the increase in DMTs available for patients with SMA, it is important to understand how caregivers view and value treatment choices [13]. The aim of this SW study was to provide estimates of the relative importance of SMA treatment attributes and drivers of preference for SMA treatment from the perspective of caregivers of pediatric patients with SMA types 1 or 2 in the United States. In this caregiver direct rating preference survey, the “risk of severe adverse events within 1 year after treatment initiation” followed by the “need for permanent ventilation within 1 year after treatment” were considered the most important treatment attributes. “Access to treatment,” “burden of treatment administration,” and other clinical improvements, including the “ability to sit without support after 1 year of treatment” and the “ability to feed orally after 1 year of treatment,” respectively, were less important attributes. Finally, the “type of facility in which the treatment is available” was the least important treatment attribute from the caregivers’ perspective.

Treatment coverage was preferred over a treatment not being covered but with payment arrangements made available as part of the “access to treatment” attribute. In terms of “burden of treatment administration,” one-time administration was preferred over continuous dosing. Caregivers preferred a one-time intravenous infusion over a one-time spinal injection, most likely because the spinal injection required the patient to receive anesthesia with the potential for adverse events related to the route of administration, while the intravenous infusion did not.

For continuous (i.e. daily, lifelong) administration attribute levels, caregivers preferred the daily oral option over the less frequent spinal injections option, suggesting that less frequent spinal injections was valued less by caregivers. In terms of the type of treatment facility, there was a preference by caregivers for a general neurology care facility closer to home over a neuromuscular specialized facility further from home.

There was a small discrepancy between the ranking and weighting of the “need for permanent ventilation within 1 year after treatment” and the “risk of severe adverse events within 1 year after treatment initiation.” Previous SW studies have avoided the risk of choice inconsistency by applying a constraint to the survey, so participants are forced to score relative to their ranking [14, 15]. However, our study allowed participants to give unrestricted weighting in which individual responses were not limited by previous ranking. This allowed for the recording and incorporation of the full range of caregiver preferences. However, in allowing for this flexibility in the survey design, issues with choice consistency, in which participants make different decisions when presented with the same choices, may have arisen. Although there is no definitive rationale for choice inconsistency in a preference survey [20], this could be related to the high level of decision complexity and outcome uncertainty [21]. Choosing health care treatments, such as those presented in this survey, is a complex task that may lead to choice inconsistency. In addition, preferences may be developed and strengthened during the completion of the survey, which may have also accounted for inconsistencies between the ranking and weighting stages of the study.

As anticipated, when presented with hypothetical treatment scenarios incorporating SW exercise preferences, caregivers preferred the scenario that incorporated the highest ranked and not the middle- or lowest-ranked treatment attribute levels. However, as with all stated preference methods, these results may not demonstrate the true preference of caregivers, as they do not reflect the real-life choices that caregivers of patients with SMA may face [22]. Treatment choices for patients with SMA are based on a range of attributes personal to the individual. Thus, caution should be employed when interpreting the results of caregivers’ preferences for treatment scenarios, as they do not account for patient clinical characteristics that would apply in clinical practice. For example, the hypothetical treatment scenarios did not account for type of SMA, age, or weight, which may mean that some treatment administration routes described in the scenarios would not be available to some individual patients. In addition, our survey was completed by a small sample size of caregivers; therefore, we were unable to examine whether the characteristics of patients with SMA (e.g., SMA types 1 or 2) had an impact on preferences for different attributes. Therefore, the interpretation surrounding preferences of hypothetical treatment scenarios should be done cautiously and only in the context of an exploratory application of study results.

In our study, we determined safety (severe adverse events) to be the most important treatment attribute to caregivers, whereas other studies did not determine this to be the most important treatment attribute [23, 24]. However, interpretation of these data should consider the wide attribute level used, ranging from 10% of patients based on the STRIVE-US complete trial [17] to a hypothetical 50% of patients, which may influence the ranking of safety attributes. It is also important to note the differences in the description of the safety attribute. Lo et al. [23] detailed significantly less severe adverse events (i.e., “risk of liver injury, and fatigue, headache, and nausea for 2 months”) compared with our study (i.e., “requiring hospitalization or events that are life-threatening or result in persistent or significant disability”). Similar to previous research, our study demonstrated that caregivers preferred better respiratory function over better motor function. Similar results for burden of treatment administration were found in another study, in which one-time treatment administration was preferred over frequent administration [23]. However, within the literature for treatment administration, other research found preference heterogeneity among caregivers in the United Kingdom, who were more likely to choose a treatment taken daily orally than a treatment administered via a spinal injection every 4 months [25].

The “burden of treatment administration” attribute demonstrated a benefit-risk hierarchy that exists for caregivers. From the ranking and weighting estimates, we can presume that convenience of treatment frequency (one time) is preferred over more frequent administration. However, within the one-time treatment administration, rankings suggest that participants may have considered spinal injections (with administration of anesthesia) riskier than intravenous administration (not requiring anesthesia). This hypothesis is supported by the results of the qualitative research where caregivers and HCPs discussed risks (anesthesia, location of injection site, and possible adverse events) with a preference toward less invasive and infrequent modes of administration, with all participants confirming that a one-time treatment is beneficial.

This is the first direct rating preference study to measure SMA treatment preferences of caregivers. The SW methodology allowed us to collect preferences with a small sample size. However, the small number of respondents did limit our ability to explore heterogeneity in preferences between caregivers, especially differences in caregiver preferences for those who care for an individual with SMA type 1 versus type 2. Therefore, there may be differences in preferences that are unaccounted for in our current estimates.

While the reference treatment described in the survey was hypothetical, the attributes and attribute levels described were based on a plausible range of values that could be experienced by the target population. Given this and the limited number of treatments available for SMA, caregivers could have associated treatment attributes to actual approved treatments, which may have influenced treatment preference choices; however, this cannot be confirmed. Moreover, the participants were reminded by the interviewer when completing the survey that the descriptions were of hypothetical rather than specific, real-world treatments and that the treatment results were not specific to any patient.

In addition, the recruitment and characteristics of caregivers included in this study may bias the results. Within the small sample size of caregivers included in the study, almost all were women, which is consistent with previous studies evaluating the caregiver experience in SMA [26]. In addition, caregiver respondents were split in terms of level of care they provided for their patients with SMA; almost half were full-time caregivers and most of the remainder were employed. Further, caregivers recruited were involved in patient organizations, and as such, tend to be highly engaged with patient care. This may not reflect the general SMA caregiver population, who vary widely in terms of demographics and the level of care they provide for their patients, which may have skewed the results of this study. Allowing for more than one parent or caregiver per family to complete the survey could have also influenced the heterogeneity across the responders.

The description of the attribute levels detailed a complex real-world treatment with interacting attributes described in the levels (i.e., adverse events were included in the treatment burden attributes). The interaction between these additional attributes in the level description, while reflecting the complex decisions that these caregivers face, may have generated estimates that do not demonstrate the true value of the attribute. Instead, these values may be related to the trade-off value of the interaction (i.e., the interaction between treatment burden may be a combination of risk and burden, not isolated to burden alone).

Although effort was made to include caregivers of patients with SMA types 1 and 2 and across age groups and all stages of the patient journey, the majority of patients had SMA type 1, and all were young (less than 2 years of age) and had received at least one SMA DMT. The over-sampling of patients with SMA type 1 may cause the results to reflect caregivers with SMA type 1 preferences. For example, a previous discrete choice experiment survey demonstrated that caregivers of patients with SMA type 1 valued improvement in breathing function more than caregivers of patients with SMA types 2 or 3 [24]. In addition, the patient population in our study reflects an earlier stage of the treatment journey during the recent SMA DMT era when age-restricted SMA gene therapies are available. In this same survey, caregivers of patients younger than 2 years of age who would potentially be eligible for SMA gene therapies had no preference for treatment age indications, whereas caregivers of patients 3–17 years old preferred treatments approved for children and adults with SMA [24]. Further, given the young age of the patient population in our study, caregivers would have only provided care and made treatment decisions for a limited period of time. However, caregiver treatment decision-making for children with chronic illnesses evolves over time due to multiple factors, including changes in disease severity [27]. Finally, all patients included in our study were from the United States, where newborn screening for SMA is part of routine care and enables earlier diagnosis, treatment, and improved therapeutic outcomes [28]. Patients in the United States also more readily have access to novel, more effective SMA DMTs compared with patients in other countries which have neither implemented early screening nor adopted new therapies [28]. Therefore, the characteristics of the patient population included in our study may also affect the generalizability of these results, particularly for caregivers of patients with SMA type 2, patients with a longer treatment journey, and/or patients outside of the United States. As a recommendation for future work, a targeted preference study focused on caregivers of patients with SMA type 2 should be performed to determine whether there are differences in treatment preferences and to account for these differences between each of the SMA types. In addition, examining caregiver treatment preferences based on the stage of the patient journey and geographic location would also be valuable. Further examination of the hierarchy of benefit-risk may be required to determine the value of the interactions separately to determine which attributes are driving choice. This could be explored through qualitative research or a think-aloud SW study in which the participant is asked to explain their decision-making process while completing the survey.

## Conclusions

This study provides a better understanding of caregiver preferences for hypothetical SMA treatment characteristics. The understanding of what caregivers perceive as important treatment attributes and the amount of risk they may be willing to accept for treatment benefit could provide important contextual information for decision-makers. Treatment administration can have a significant impact on caregivers. Both the quantitative and qualitative component of the study found that one-time intravenous infusion was preferred to all other administration options, with the benefits of less risk, less frequent administration, and less invasiveness all cited as reasons for this preference over other options, such as spinal injection. The valuation of the hypothetical treatments identified the burden of treatment administration as the driving force of the ranking of treatments. The results of this study can be used to promote better medical decision-making for caregivers of patients living with SMA and help promote patient-centered care.

## Supporting information

S1 File (DOCX)
