# Supplementary material for: Treatment preferences in spinal muscular atrophy: A swing weighting study for caregivers of patients with SMA types 1 and 2
Source: PLoS One. 2024 Oct 21;19(10):e0309666. doi: 10.1371/journal.pone.0309666 (PMC11493260; doi:10.1371/journal.pone.0309666)
Supplement: S1 File — (DOCX) [file pone.0309666.s001.docx]

**SUPPORTING INFORMATION**

Appendix 1. Attributes elicitation phase and pilot phase

Appendix Table S1. Literature search criteria

# **Appendix 1. Attributes elicitation phase and pilot phase**

**Literature search**

A targeted literature search identified studies that examined patient satisfaction or preferences in the context of treatment for neuromuscular or motor neuron conditions. The aim was to obtain information regarding the characteristics that were mentioned or assessed with regards to preference for treatment. These studies were then analyzed to identify the reported treatment characteristics. This review was not intended to be systematic or exhaustive, but was nevertheless based on a rigorous search strategy implemented in the Medline and Embase databases via Ovid interface and in the Professional Society for Health Economics and Outcomes Research (ISPOR) scientific presentations database utilizing the criteria outlined in **Appendix Table S1**.

**Appendix Table S1. Literature search criteria**

| **PICOS Criteria** | |
| --- | --- |
| Population | Patients with SMA or other pediatric neuromuscular diseases |
| Interventions | Any |
| Comparator | Any |
| Outcome | Preferences, treatment satisfaction, or treatment characteristics as perceived by patients with SMA or their caregivers |
| Study design | Any |
| **Additional criteria** | |
| Publication year | 2009–2019 |
| Language | English |
| Geography | Any |

PICOS, Population, Interventions, Comparator, Outcome, Study Design; SMA, spinal muscular atrophy.

From 104 screened articles, a total of five final publications were included and data were extracted and summarized. Of the five publications, three were stated preference studies and two were qualitative research. The review allowed identification of several potential attributes. All treatment features cited in the selected publications as potentially having an impact on patient preferences or clinician choices were collected. These features were grouped into 10 categories based on their nature.

**Interviews**

To identify salient treatment attributes, interviews with four caregivers of children (aged 3–15 years) who received disease-modifying treatments (DMTs) for spinal muscular atrophy (SMA) and four health care professionals (HCPs) (three neurologists and one nurse specializing in pediatric neurology) with experience in SMA treatment were completed. All HCPs were required to have treated at least two patients with SMA types 1 or 2 in the past 12 months. Caregivers interviewed were required to be either the main caregiver or co-main caregiver of a patient with SMA types 1 or 2. The caregivers’ interview guide included questions about the child’s diagnosis, available treatments for SMA, and their experiences with SMA treatments (route and frequency of administration, efficacy, adverse events [AEs], accessibility, satisfaction with treatment results). The last task for the caregivers was to rate all treatment characteristics that were mentioned in the discussion, starting from the one they believed to be most important. The interview guide for the HCPs included questions about their professional experience, number of SMA patients treated, and clinical experience with the three approved DMTs (burdens and benefits). HCPs were also asked to select up to 12 of the biggest drivers for choosing a SMA treatment that are important first from their perspective, and second, to do the same task from caregivers’ perspective.

Caregivers primarily considered treatment efficacy (increasing chances of survival and improving patients’ independence), burden of administration (invasiveness of administration, total time spent on activities related to treatment administration [including travel]), long-term safety, and accessibility of therapy (time required to obtain the first dose). HCPs primarily focused on effectiveness (improvement in motor, respiratory, or bulbar function, or slowing of disease progression) and availability/quality of evidence (clinical trial data or personal observation), but also considered invasiveness of administration and accessibility of treatment (ease of obtaining reimbursement). As a conclusion from phase 1, the most important characteristics of DMTs for treating SMA types 1 and 2 according to caregivers and HCPs were effectiveness, burden of administration, and accessibility.

**Pilot phase**

The objective in the pilot phase was to ensure that participants were able to answer the survey without problems and to understand the swing weighting (SW) exercise. Screening and sociodemographic questions were developed as a first part of the online survey to ascertain that the respondents met study criteria. In addition, there was a section with questions about the patient for whom they care, such as age, time since diagnosis, physical functioning, treatments received, etc. After the survey was completed by teleconference application, an interview followed based on a specific interview guide to ensure that respondents understood the vocabulary and the choice task and that the burden of completion was acceptable.

The 2-wave pilot survey was moderator-assisted and completed by five caregivers (four parents of children with SMA type 1, one parent of a child with SMA type 2). In each wave, the respondents were asked to assess its complexity and their overall experience after completing the survey. The results of the interviews were summarized and analyzed by the research team, and modifications to the questionnaire were introduced to further improve clarity of the SMA treatment characteristics (supplementary clarifying information was added in a study information sheet, in the description of attributes and levels, and in the instructions of SW tasks; four sub-tasks concerning the attributes containing numerical changes in levels were removed from the SW exercise to reduce the cognitive burden of the survey). All caregivers confirmed that the attributes we presented in the survey were very important characteristics of SMA treatments and that these attributes described patient quality of life well.

Caregivers were asked how they made their choices in the SW exercises. For burden of treatment administration, for example, all expressed preference of one-time intravenous infusion over a spinal injection approach, regardless of whether the spinal injections were one-time or continuous. The reasons for this preference included the belief that that spinal injection was riskier because it required going under anesthesia; that it had to be done in the spine, which could increase the risk of AEs; that the procedure itself was intimidating (potential scar tissue issue and need for local anesthesia); that it may be impossible to give a spinal injection in cases where the child has a curved spine; and that a less invasive and less frequent way of administration was preferable. They all confirmed that the idea of one-time treatment was extremely beneficial.

**Levels elicitation**

The elicitation of attributes and levels that were used in SW exercises was based on a literature review, interviews with caregivers and HCPs, and clinical trial data available by 2021. **Table 1** in the manuscript represents a summary of the sources used to develop the attributes and levels used in the SW exercise. The levels of the four attributes, ability to sit without support, need for permanent ventilation, ability to feed orally, and risk of severe adverse events, were developed based on clinical trial data of the three approved SMA treatments for symptomatic patients with SMA types 1 and 2 treated shortly after diagnosis in 2021: nusinersen, onasemnogene abeparvovec, and risdiplam.
